# YAP1 plays a key role of the conversion of normal fibroblasts into cancer-associated fibroblasts that contribute to prostate cancer progression

**DOI:** 10.1186/s13046-020-1542-z

**Published:** 2020-02-17

**Authors:** Tianyu Shen, Yang Li, Shimiao Zhu, Jianpeng Yu, Boya Zhang, Xuanrong Chen, Zheng Zhang, Yuan Ma, Yuanjie Niu, Zhiqun Shang

**Affiliations:** Tianjin Institute of Urology, The Second Hospital of Tianjin Medical University, Tianjin, 300211 China

**Keywords:** Yes-associated protein 1 (YAP1), Cancer-associated fibroblasts (CAFs), Normal fibroblasts (NFs), Prostate cancer (PCa)

## Abstract

**Background:**

Cancer-associated fibroblasts (CAFs) are an important part of the tumour microenvironment, and their functions are of great concern. This series of experiments aimed to explore how Yes-associated protein 1 (YAP1) regulates the function of stromal cells and how the normal fibroblasts (NFs) convert into CAFs in prostate cancer (PCa).

**Methods:**

The effects of conditioned media from different fibroblasts on the proliferation and invasion of epithelial cells TrampC1 were examined. We then analysed the interaction between the YAP1/TEAD1 protein complex and SRC, as well as the regulatory function of the downstream cytoskeletal proteins and actins. A transplanted tumour model was used to explore the function of YAP1 in regulating tumour growth through stromal cells. The relationship between the expression of YAP1 in tumour stromal cells and the clinical characteristics of PCa patients was analysed.

**Results:**

The expression level of YAP1 was significantly upregulated in PCa stromal cells. After the expression level of YAP1 was increased, NF was transformed into CAF, enhancing the proliferation and invasion ability of epithelial cells. The YAP1/TEAD1 protein complex had the capability to influence downstream cytoskeletal proteins by regulating SRC transcription; therefore, it converts NF to CAF, and CAF can significantly promote tumour growth and metastasis. The high expression of YAP1 in the tumour stromal cells suggested a poor tumour stage and prognosis in PCa patients.

**Conclusion:**

YAP1 can convert NFs into CAFs in the tumour microenvironment of PCa, thus promoting the development and metastasis of PCa. Silencing YAP1 in tumour stromal cells can effectively inhibit tumour growth.

## Background

Prostate cancer (PCa) is one of the most common causes of cancer death in the world with the highest incidence rate and the second highest mortality rate among the male population in the United States in 2018 [1].

The Hippo signalling pathway plays an important role in the development of prostate cancer [2–5]. The Hippo signalling pathway contains 13 core proteins including MST1 / 2, SAV1, LATS1 / 2, MOB1A, MOB1B, YAP1, TAZ and TEAD1–4 [6]. YAP1 acts as a downstream transcriptional coactivator of the Hippo pathway. Its abnormal expression causes malignant proliferation and metastasis, induces epithelial-mesenchymal transition, and produces possible cancer drug resistance [7–9]. In addition, since YAP1 is active in cancer cells, it can regulate a variety of cancer genes or form complexes with them and then jointly regulate the downstream target genes.

The impact of the tumour microenvironment (TME) on cancer has recently drawn much attention [10, 11]. As one of the key components of the tumour microenvironment, cancer-associated fibroblasts (CAFs) play a significant role on tumour progression and metastasis [12, 13]. The activation of normal fibroblasts into CAFs can result in the secretion of abundant tumour-promoting factors and facilitate the malignant behaviour of tumour cells through a complicated paracrine signal network. For example, CAFs rely on the activation of HIF-1 to secrete carbonic anhydrase (CAIX), which raises the acidity of extracellular matrix; CAFs secrete MMP-2/9 [14], therefore inducing the epithelial-mesenchymal transition (EMT) in tumour cells and enhancing the migration ability of tumour cells [15]. We aimed to discover the regulatory function of YAP1 in PCa related fibroblasts cells as well as the association between YAP1 and the conversion from NF to CAF in PCa.

This study found that in prostate cancer stromal cells, YAP1, FAP and α-SMA expression levels were significantly elevated compared to those of normal cells. We further conclude that due to the high expression of YAP1, normal fibroblasts are activated into CAFs. During this process, SRC is regulated by the YAP1/TEAD1 complex, which leads to the activation of downstream actins and cytoskeletal proteins. After conversion, CAFs significantly enhance the proliferation and invasion of tumour epithelial cells. We believe that, as one of the indicators of the biological behaviour of malignant tumours, YAP1 is increased in the stroma of the PCa and there could be a certain reference value for the diagnosis of cancer. This may indicate that the disease progresses to an advanced stage or even metastasizes. As a result, detecting the expression level of YAP1 in prostate cancer stromal cells may be an early indicator for the disease prognosis. YAP1 may be used as a potential target for new targeted cancer therapy.

## Materials and methods

### Ethical approval for the study protocol

This study was approved by the Ethics Committee of the Second Hospital of Tianjin Medical University, Tianjin, China. (No. KY2019K077 and No. YN2019Y70). Written informed consent was obtained from all patients, and the study was conducted in accordance with the Declaration of Helsinki.

### Human samples

The prostate tissue specimens that were used in this study were surgical specimens from PCa patients with complete clinicopathological data. Benign prostatic hyperplasia tissues were acquired by transurethral resections of the prostate (*n* = 12), and prostate cancer tissue specimens (*n* = 25) were acquired by radical prostatectomy. These samples were paraffin-embedded and subjected to IHC and IF assays.

### Antibody

The following antibodies were used in this study for western blot, immunohistochemistry, immunofluorescence staining and immunoprecipitation: YAP1 (Santa Cruz Biotechnology, sc-376,830, 1:100 dilution for western blot; 1:50 dilution for immunohistochemistry and 1:50 dilution for Immunofluorescence staining; Abcam, ab52771, 1:20 dilution for IP), p-YAP1 (Abcam, ab76252, 1:10000 dilution for western blot), α-SMA (Abcam, ab5694, 1:200 dilution for western blot; 1:100 dilution for immunohistochemistry and 1:100 dilution for immunofluorescence staining), FAP (Abcam, ab53066, 1:1000 dilution for western blot and 1:100 dilution for immunofluorescence staining), SRC (Signalway Antibody, #40790, 1:1000 dilution for western blot, 1:100 dilution for immunohistochemistry and 1:100 dilution for immunofluorescence staining), p-SRC (Abcam, ab4816, 1:1000 dilution for western blot), TEAD1 (Abcam, ab133533, 1:20 dilution for IP and 1:500 dilution for western blot), GAPDH (Sungene Biotech, KM9002, 1:5000 dilution for western blot).

### Cell culture and cell lines

The prostate and prostate cancer cell lines that were used in this experiment, including TrampC1, RM1, CAF and NF, were all derived from Dr. Chang, George Whipple Lab for Cancer Research, and these four types of cells are of mouse origin [16, 17]. TrampC1 and RM1 were cultured in RPMI-1640 medium (Gibco, Waltham, MA USA) containing 10% foetal bovine serum (Gibco, Waltham, MA USA) and culture conditions of 37 °C with 5% CO_2_. CAF and NF were cultured in DMEM (Gibco, Waltham, MA USA) containing 10% foetal bovine serum (Gibco, Waltham, MA USA) and incubated at 37 °C with 5% CO_2_.

In this experiment, human prostate cancer hCAF and human prostate hNF were taken from the primary culture of urological surgical specimens from the Second Hospital of Tianjin Medical University. The hCAF and hNF samples were cultured in DMEM (Gibco, Waltham, MA USA) containing 10% foetal bovine serum (Gibco, Waltham, MA USA) and incubated at 37 °C with 5% CO2.

### MTT assay

A 5 g/mL concentration of MTT solution was prepared and stored at 4 °C in the dark. The cells to be tested were seeded in a 96-well plate (cell number 2 × 10^3^ cells/well, medium 100 μL/well). The samples were incubated for 3–6 days at 37 °C with 5% CO2. Then, 50 μL of MTT solution were added to each well and incubated at 37 °C for 4 h. The supernatant was aspirated, and 150 μL of DMSO were added to each well and shaken on a plate shaker. The microplate reader measures the optical density OD value of each well at a wavelength of 570 nm. The value-added active fold lines were drawn using GraphPad Prism 5 software (GraphPad Software, La Jolla, CA, USA).

### Western blot

Total cellular proteins were extracted using RIPA (Thermo Scientific, 89,901). The prepared protein sample was added to the gel lane of the separation gel, and electrophoresis was performed using a constant voltage. After the end of the electrophoresis, the protein was transferred using a PVDF membrane. After the completion of the electroporation, the PVDF membrane was sealed with skim milk for 60 min. After TBST was washed, the primary antibody was incubated overnight at 4 °C. The primary antibody was washed away by TBST, and the secondary antibody solution corresponding to the primary antibody was added and incubated at room temperature for 1 h. The secondary antibody was washed with TBST and prepared for exposure. The prepared developing solutions A and B are mixed in proportion (Immobilon Western, Chemiluminescent HRP Substrate, Millipore Corporation, Billerica, MA, USA), and the mixed liquid is dropped on the corresponding molecular weight strip of the PVDF film and placed in an exposure machine for exposure.

### Immunohistochemistry

After the specimen was fixed with a formalin solution, wax block preparation was performed and anti-separation sections prepared. Dewaxing was performed for water and antigen retrieval by conventional methods. Then, 3% H_2_O_2_ was added to the specimen to remove the endogenous peroxidase in the specimen. After washing with PBS, the primary antibody was added dropwise and incubated at 4 °C for 12–18 h. After rewarming, the secondary antibody was added to the specimen and incubated at 37 °C for 30 min. After the PBS was washed again, the pre-configured DAB solution was added dropwise, and after the staining was completed, the sections were rinsed in PBS buffer in time. After washing with tap water, the nucleus was counterstained by adding haematoxylin working solution. The results were observed under a microscope, the positive rate was counted, and the results were analysed.

### Transwell invasion assay

Matrigel was pre-treated to 4 °C until liquified. Matrigel was diluted with pre-cooled serum-free 1640 medium (the dilution ratio was 1:3), and 60 μL of the diluted gel were added to a 24-well plate (Corning Costar, 3524) in a Transwell chamber (BD FALCON, 353097) for 6 h. The cells were suspended in serum-free medium. A total of 10^5^ cells were added to each Transwell upper chamber, and 500 μL of foetal bovine serum-containing medium or conditioned medium were placed in the Transwell lower chamber. The cell culture was performed as described above. After 24 h, the cells on the upper surface of the Transwell membrane were removed with a cotton swab, and the cells on the lower surface of the Transwell membrane were fixed and stained. The cells on each Transwell membrane were photographed and counted. The statistical results were drawn.

### In vitro transfection

The following shRNA plasmids were used in this study for in vitro transfection: YAP1 Mouse shRNA Plasmid, CAT#: TG502437, Origene; TEAD1 Mouse shRNA Plasmid, CAT#: TL513813, Origene; shRNA vector, CAT#: TR30007, Origene; YAP1 Mouse Tagged ORF Clone, CAT#: MR226049, TrueORF®; and TEAD1 Mouse Tagged ORF Clone, CAT#: MR206462, TrueORF®. The siRNA sequences (Supplementary Table 1) were synthesized by RiboBio (Guangzhou, China), and a scrambled siRNA (RiboBio) was used as the negative control. The cells were transfected with liposomes. The plasmid was mixed with Transfection Reagent 1:1–1:4 and added to opti-MEM for 30 min. The above mixture was added to the medium of the cells. The new medium was replaced after 24–48 h. The transfected cells were screened using G418. A stable transfected cell line was finally obtained.

### Immunofluorescence staining

The cells were seeded in glass slides and treated with paraformaldehyde and Triton, and the primary antibody was incubated overnight at 4 °C. After the secondary antibody was added dropwise, it was incubated at room temperature for 1 h, and after washing with PBS, the nuclei were stained with DAPI. The photograph was taken under an Olympus FV1000D confocal microscope [18].

### RNA isolation and quantitative RT-PCR analysis

The total RNA was extracted using Trizol reagent according to the manufacturer’s protocol. The RNA was reverse transcribed using a reverse transcription kit (RevertAid First Strand cDNA Synthesis Kit, Thermo Scientific, Waltham, MA USA) to obtain cDNA. The mRNA reverse transcription-PCR (RT-PCR) primers for YAP1, α-SMA, FAP, SRC, MYL9, F-actin and paxillin were purchased from Applied Biosystems. The primer sequences are shown in Supplementary Table 2. The expression of the mRNAs in the quantitative RT-PCR analysis was determined by an Applied Biosystems 7900 Real Time PCR System (Thermo Scientific, Waltham, MA USA). Small nucleolar RNA U6 was used as an internal reference for normalization.

### Co-immunoprecipitation (co-IP)

A small amount of cell lysate was used as the input. The remaining lysate was added to the YAP1 or TEAD1 antibody and placed on a shaker at 4 °C overnight. The pretreated protein A agarose magnetic beads were added to the lysate to fully couple the YAP1 or TEAD1 antibody to the protein A agarose magnetic beads. After the immunoprecipitation reaction, the agarose beads were collected. The agarose beads were washed 3 times with the lysis buffer. The SDS loading buffer was added to the liquid and heated at 95 °C for 5 min. Analysis was performed using western blot.

### Chromatin immunoprecipitation (CHIP)

The prepared cells were subjected to the YAP1 or TEAD1 CHIP assay using the EpiQuik Chromatin Immunoprecipitation Kit (Epigentek, Farmingdale, NY, USA) according to the protocol [18]. PCR was performed using primers specific for the YAP1 or TEAD1 binding regions in the SRC promoter. The primer sequences of the promoter region are shown in Supplementary Table 2.

### Luciferase reporter assays

Luciferase reporter constructs (MCS-firefly_Luciferase and TK promoter-Renilla_Luciferase) were processed by Genechem (Shanghai Co., Ltd.). The 293 T cells were transfected with the SRC luciferase reporter constructs (MCS-firefly_Luciferase) with or without YAP1 and TEAD1 overexpressing plasmid. Luciferase activity was normalized to Renilla luciferase activity. A Dual-Luciferase Reporter Assay System (Promega) was applied to measure the luciferase value according to the manufacturer’s instructions [18]. Three independent assays were performed in triplicate.

### In vivo experiments

The animal studies were approved by the Second Hospital of Tianjin Medical University, Tianjin, China. Male nude mice (6 weeks old, *n* = 12) were purchased from Beijing HFK Bioscience Co. Ltd. (Beijing, China). The animal studies were approved by Tianjin Institute of Urology, Tianjin, China. Male nude mice (6 weeks old, *n* = 20) were purchased from Beijing HFK Bioscience Co. Ltd. (Beijing, China). Subcutaneous tumour growth assays were performed with CA, CAFshYAP1, NF and NFoverexpressYAP1 stable cell lines. We mixed each of the four stable cell lines previously described in the paper with the epithelial cell TrampC1 at a ratio of 1:1 (1 × 10^6^ stromal cells mixed 1 × 10^6^ epithelial cells) to obtain 4 different experimental groups: CAF TrampC1, CAFshYAP1 TrampC1, NF TrampC1, and NFoverexpressYAP1 TrampC1. Five BALB/c nude mice are in each group. After 2 weeks, 20 of the injected mice developed tumours. The tumours were harvested under standard institutionally approved processes. The tumour samples were paraffin fixed and processed for IHC analysis.

### Statistical analysis

SPSS 22 statistical software (SPSS, IBM Corporation, Armonk, NY, USA) was used for the statistical analysis. A one-way analysis of variance was used for multiple comparisons. A paired t-test was used for comparison between different treatment groups and control groups. GraphPad Prism 5 software was used to draw the graphics. *P* < 0.05 indicates a statistically significant difference in results. *P* < 0.05 was marked as *, *P* < 0.01 was marked as **, *P* < 0.001 was marked as ***, and no significant difference was expressed as n.s.

## Result

### Increased expression of YAP1 in stromal cells in PCa

There are many reports that YAP1 is upregulated in prostate cancer epithelial cells. YAP1 can bind to androgen receptor (AR) and affect the proliferation of prostate cancer epithelial cells, thus affecting the progression of prostate cancer [19]. However, the mechanism of action of YAP1 in prostate cancer stromal cells is not clear.

**Fig. 1:**
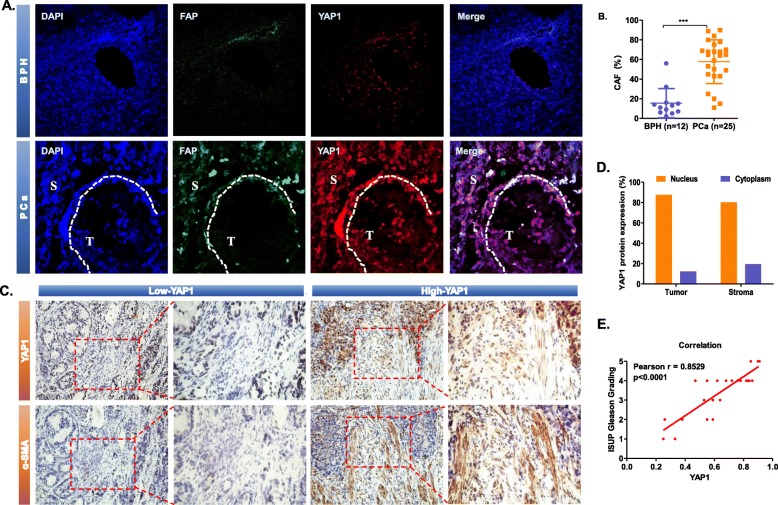
Increased expression of YAP1 in stromal cells in PCa. **a** Immunofluorescence staining showed the protein expression level and location of FAP and YAP1 in BPH (*n* = 12) and PCa (*n* = 25). FAP was displayed in red and YAP1 was displayed in green. The nuclei were stained with DAPI and are shown in blue. The representative image had a magnification of 400 x. **b** Statistical results show that the proportion of CAFs in PCa tissues (*n* = 25) is significantly higher than that in BPH tissues (*n* = 12), *p* < 0.0001. **c** Immunohistochemical staining showing the expression level and location of YAP1 and α-SMA in PCa (*n* = 25). Based on the expression of YAP1 in stromal cells, the IHC results were divided into two groups: Low-YAP1 (*n* = 9) and High-YAP1 (*n* = 16). The representative image had a magnification of 200 x. **d** Statistical results showing the localization of YAP1 in the cells of the “YAP1-High” group (*n* = 16). YAP1 was localized in the nucleus in 87.65% of tumour cells and 80.36% of stromal cells, while it was localized in the cytoplasm in 12.35% of tumour cells and 19.64% of stromal cells. **e** The correlation between YAP1 expression in prostate cancer stromal cells and the Gleason Grading. The abscissa represents the positive rate of YAP1 in prostate cancer stromal cells, and the ordinate represents the Gleason Grading. Pearson *r* = 0.8529, *p* < 0.0001

Our group selected 37 clinical patient specimens, including 12 from benign prostatic hyperplasia (BPH) patients and 25 from PCa patients. The paraffin sections of each specimen were immunofluorescence double stained, and representative pictures were selected for the shown figure. We used a specific marker of CAF, fibroblast activation protein (FAP), to localize CAF [20]. To understand the expression of YAP1 protein in tumour epithelial cells and stromal cells, we consulted pathologists to identify the tissue morphology. According to the opinions of pathologists from the Second Hospital of Tianjin Medical University, we distinguished stromal cells (S) and tumour epithelial cells (T) based on the pathological structure, as shown in the figure (Fig. 1a). We found that in BPH tissues, YAP1 and FAP were underexpressed in stromal cells. In PCa tissue, YAP1 was significantly upregulated in stromal cells, and FAP was also increased in stromal cells. Furthermore, YAP1 is also highly expressed in epithelial cells in PCa tissues. Therefore, we believed that there was a high expression of YAP1 in both epithelial cells and stromal cells in prostate cancer tissues. However, this article focuses on the function of YAP1 protein in stromal cells. We found that the expression level of FAP in PCa stromal cells was significantly higher than that in BPH. This indicated that there was a large amount of CAF in PCa tissues, while CAF was almost absent in BPH tissues. More noticeably, there was a large overlap between the staining regions of YAP1 (red fluorescence) and FAP (green fluorescence) in stromal cells. This indicated that both YAP1 and FAP were highly expressed in stromal cells, suggesting that YAP1 was highly expressed in CAF. The statistical results again show that the CAF content in PCa (*n* = 25) tissues is significantly higher than that in the BPH (*n* = 12) tissues (Fig. 1b) (*p* < 0.0001). This led us to speculate that the number of CAFs was increased in PCa due to the high expression of YAP1. Next, we selected alpha-smooth muscle actin (α-SMA) as another specific marker of CAFs for immunohistochemical (IHC) staining of the above 25 PCa specimens. Based on the expression of YAP1 in the stromal cells, we divided the IHC results into two groups: Low-YAP1 (*n* = 9) and High-YAP1 (*n* = 16). From the pictures, we can see that when YAP1 was underexpressed in stromal cells, the expression of α-SMA was also low (Fig. 1c), indicating that the number of CAFs was small. By contrast, the “High-YAP1” group suggested that α-SMA expression was significantly elevated when YAP1 was upregulated in stromal cells, indicating that the high expression of YAP1 in stromal cells promoted CAF formation. In addition, the expression of YAP1 in stromal cells also positively affected the expression level of YAP1 in epithelial cells. In addition, we performed a statistical analysis of the IHC results. The results showed that in the High-YAP1 group, YAP1 was expressed in the nucleus of 87.65% of tumour cells, while YAP1 was expressed in the nucleus of 80.36% of stromal cells. This indicated that YAP1 was in an activated state (Fig. 1d). According to the definition of Gleason Grading formulated by the International Society of Urological Pathology (ISUP) in 2014 [21], we calculated the correlation between YAP1 expression in prostate cancer stromal cells and Gleason Grading (Fig. 1e). In 25 PCa specimens, the high expression of YAP1 in prostate cancer stromal cells was positively correlated with the Gleason Grading (*R* = 0.8529, *P* < 0.0001).

The table shows that the expression of YAP1 in the stromal cells of prostate cancer patients is positively correlated with initial PSA (Table 1). Patients with high expression of YAP1 in stromal cells tend to have a malignant tumour grade and stage. Not only will lymph node metastasis occur, but seminal vesicle metastasis may also occur.

**Table 1:** Clinical Feature

Variables	All *n*=25	YAP1		*P* value^#^
		Low *n*=9	High *n*=16	
Age				
<70		3	4	0.67
≥70		6	12	
iPSA				
≤10		7	3	0.01^*^
>10		2	13	
Tumor stage				
T2		7	4	0.02^*^
T3/T4		2	12	
ISUP Gleason Grading				
≤3		6	3	0.03^*^
>3		3	13	
Lymph node metastasis				
No		8	5	0.01^*^
Yes		1	11	
Seminal vesicle metastasis				
No		8	6	0.03^*^
Yes		1	10	

#*P* value was analyzed by Chi-square test; * indicates *P*<0.05 with statistical significance; iPSA means initial PSA

CAF and NF immortalized cell lines were used for further research. These two mouse-originated cell lines were gifted by Dr. Chang, George Whipple Lab for Cancer Research. First, we examined the mRNA and protein levels of α-SMA, FAP, and YAP1 in CAFs and NFs (Supplementary Figure S1A-B) to confirm that CAFs have a higher expression of α-SMA, FAP and YAP1. This completed the identification of the selected cells. From the immunofluorescence double staining (Supplementary Figure S1C), YAP1 was mainly expressed inside the nucleus, and α-SMA was expressed in the cytoplasm in both CAFs and NFs.

### YAP1 plays an important role in the conversion of NFs to CAFs in vitro

**Fig. 2:**
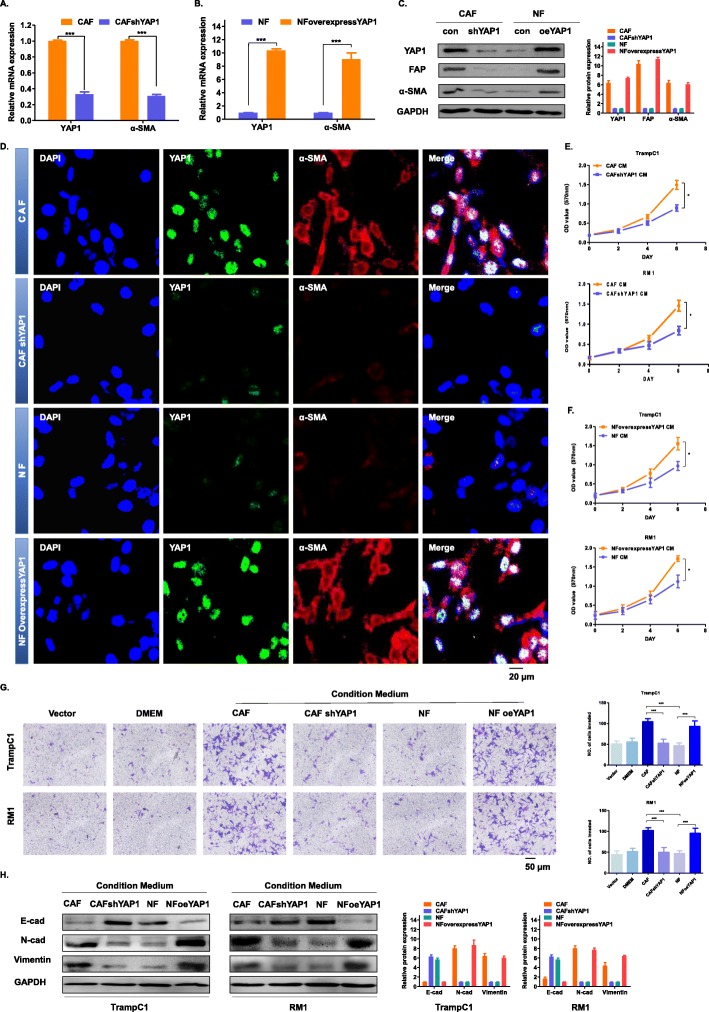
YAP1 plays an important role in the conversion of NFs to CAFs in vitro. **a**-**b** The mRNA expression of YAP1 and α-SMA in the CAF, CAFshYAP1, NF and NFoverexpressYAP1 groups were detected by qRT-PCR. **c** The protein expression of YAP1, FAP and α-SMA in the indicated four cell lines were detected by western blot. GAPDH was used as an endogenous reference gene. **d** Immunofluorescence staining shows the expression level and location of YAP1 and α-SMA in the four indicated four cells. The nuclei were stained with DAPI. The representative image had a magnification of 400 x. **e**-**f** The MTT experiment showing the effect of the conditioned medium on the four indicated cell lines on the proliferation of the epithelial cells TrampC1 or RM1. The absorbance value was detected at a wavelength of 570 nm (*P < 0.05). **g** The Transwell invasion assay detects the effect of the conditioned medium on the indicated four cell lines on the invasive ability of the epithelial cells TrampC1 or RM1. Statistical results (right side) of the above invasive ability. Five visual field counts were taken for each group, and the ordinate indicates the number of invading cells (***P < 0.001). **h** The protein expression of E-cad, N-cad and vimentin in the indicated four cell lines were detected by western blot. GAPDH was used as an endogenous reference gene

To further investigate the mechanism of action of YAP1 in the formation of CAFs, we constructed two new stable cell lines using plasmids, named CAFshYAP1 and NFoverexpressYAP1. In the subsequent experiments, four cell lines CAF, CAFshYAP1, NF and NFoverexpressYAP1 were simultaneously tested. After establishing a stable cell line, we examined the mRNA expression levels of YAP1 and α-SMA in the four cell lines mentioned above (Fig. 2a-b), in addition to the protein expression levels of YAP1, FAP and α-SMA (Fig. 2c). Interestingly, the expression level of α-SMA in the CAFs declined as YAP1 declined, and the expression level of α-SMA increased in the NFs as YAP1 increased. In all four types of cells, immunofluorescence staining showed that YAP1 was distributed in the nucleus and α-SMA was distributed in the cytoplasm (Fig. 2d). Additionally, the expression level of α-SMA was regulated by YAP1. Therefore, the increased YAP1 resulted in an increase in CAFs. In conclusion, the expression of YAP1 may affect the mutual conversion of CAF and NF. In other words, once YAP1 is reduced in the CAFs, CAFs may revert to NFs; once YAP1 is increased in the NFs, the NFs may be converted to CAFs.

We used siYAP1 and the inhibitor verteporfin (VP) to reduce the activity of YAP1 in CAFs (Supplementary Figure S2A-B), and we then found that the proliferation ability of the CAFs was significantly inhibited (Supplementary Figure S2C-D) and that when the YAP1 level was raised in the NFs (Supplementary Figure S2E), their proliferation ability was significantly enhanced (Supplementary Figure S2F). Thus, it is confirmed that YAP1 has a regulatory effect on the proliferation of CAFs.

We further explored whether YAP1 can affect the proliferation and invasion of epithelial cells through mesenchymal cells [22, 23]. To explore the effects of the conditioned medium of fibroblasts on tumour cells, we selected two prostate cancer epithelial cells, TrampC1 and RM1, for experiments. We found that when the level of YAP1 was decreased in the CAFs, the proliferation of TrampC1 and RM1 was attenuated (Fig. 2e). When YAP1 was increased in the NFs, the proliferation of TrampC1 and RM1 will also be enhanced (Fig. 2f). Additionally, due to the upregulation of YAP1, the conditioned medium of the fibroblasts promoted the invasion of the above two tumour cells (Fig. 2g). We examined TrampC1 and RM1 after treatment with the fibroblast-conditioned medium and found that the expression of E-cadherin was decreased in the prostate cancer epithelial cells and that the expression of N-cadherin and vimentin was increased due to the upregulation of YAP1 (Fig. 2h). This indicates that the conditioned medium of fibroblasts that highly express YAP1 promotes the epithelial-mesenchymal transition (EMT) of the above two tumour cells.

### YAP1/TEAD1 protein complex activates cytoskeletal proteins to transform NFs to CAFs by regulating SRC

**Fig. 3:**
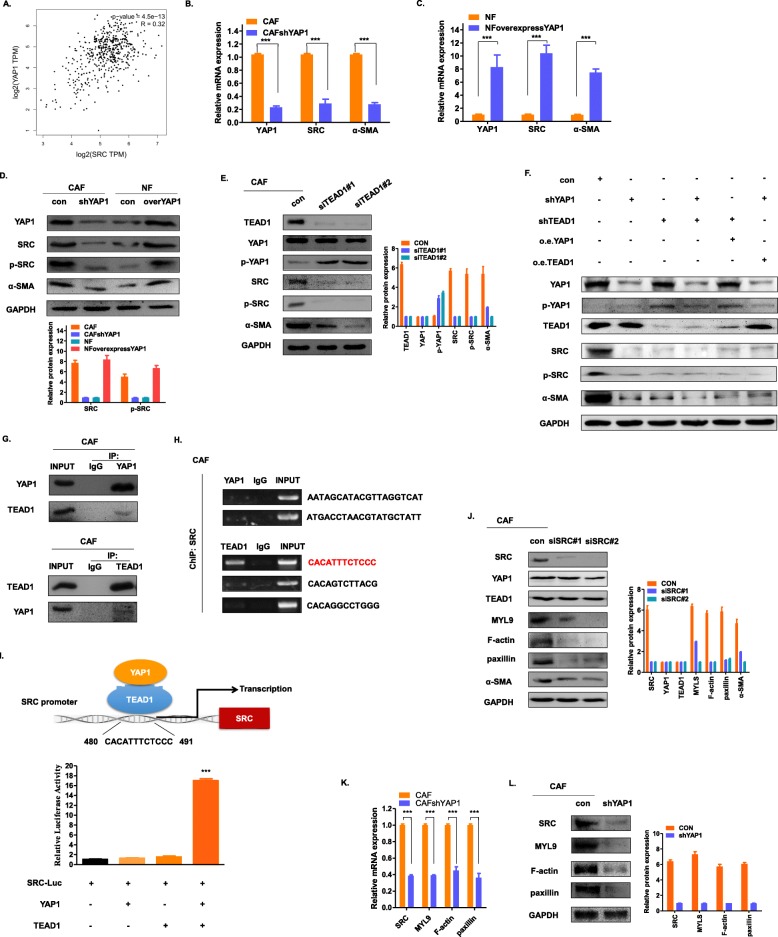
YAP1 activates actin and cytoskeletal proteins to transform NFs to CAFs by regulating SRC. **a** The association of YAP1 and SRC in prostate cancer was analysed online at http://gepia.cancer-pku.cn/. Pearson R = 0.32. **b**-**c** The mRNA expression levels of YAP1, α-SMA and SRC in the indicated four cell lines were detected by qRT-PCR. **d** The protein expression levels of YAP1, α-SMA, SRC and p-SRC in the indicated four cell lines were detected by western blot. GAPDH was used as an endogenous reference gene. **e** Western blot was used to detect the protein expression levels of TEAD1, YAP1, p-YAP1, SRC, p-SRC and α-SMA after siTEAD1 transfection of the CAFs. GAPDH was used as an endogenous reference gene. **f** Western blot was used to detect the protein expression levels of TEAD1, YAP1, p-YAP1, SRC, p-SRC and α-SMA when they were knocked down or overexpressed in CAFs. GAPDH was used as an endogenous reference gene. **g** The interaction between YAP1 and TEAD1 in the CAFs was determined by the co-IP assay. The relative levels of YAP1 or TEAD1 in these cells were determined by western blot using a YAP1 or TEAD1 antibody. **h** Chromatin immunoprecipitation (ChIP) of the CAFs was performed with control IgG and TEAD1 antibodies. The precipitation of the SRC promoter was examined by PCR. **i** A dual-luciferase reporter assay driven by the SRC promoter was co-transfected in the presence or absence of YAP1 or TEAD1. The relative luciferase activities were determined by calculating the ratio of firefly luciferase activities over Renilla luciferase activities. Three independent experiments were conducted, with the means±s.d. of the relative luciferase activities shown. **j** Western blot was used to detect the protein expression levels of SRC, YAP1, TEAD1, MYL9, F-actin, paxillin and α-SMA after siSRC transfection of the CAFs. GAPDH was used as an endogenous reference gene. **k** qRT-PCR detection of mRNA expression levels of MYL9, F-actin and paxillin in the CAFshYAP1 group. **l** Western blot was used to detect the protein expression levels of SRC, MYL9, F-actin and paxillin in the CAFshYAP1 group. GAPDH was used as an endogenous reference gene

We have proved that YAP1 was associated with the conversion of NFs into CAFs, but its mechanism remains unclear. SRC protein could regulate a series of actins and cytoskeletal proteins [24–27] that are required for the CAF to maintain its own phenotype [28–30]. Using GEPIA (http://gepia.cancer-pku.cn/) [31], we confirmed that there is a positive correlation between YAP1 and SRC expression in prostate cancer (Fig. 3a). According to The Cancer Genome Atlas (TCGA), high SRC expression suggests a poor prognosis of PCa patients (Supplementary Figure S3A). We verified that in the above four cell lines, when YAP1 was knocked down in CAFs, SRC decreased; after YAP1 was overexpressed in NFs, SRC increased (Fig. 3b-d). Interestingly, a decrease in YAP1 also resulted in a decrease in the phosphorylation level of SRC, rendering the SRC inactive (Fig. 3d).

According to known reports, YAP1 cannot bind directly to DNA. However, when YAP1 acts as a transcriptional cofactor, it combines with transcription factors to mediate the transcription of downstream genes [32]. The transcription factor TEAD1 is a common binding molecule of YAP1 [33]. The TEAD1 N-terminal TEA DNA binding domain binds to the C-terminal region of YAP1 [34, 35]. After binding to TEAD1, YAP1 relies on the DNA binding domain of TEAD to initiate downstream gene transcription [32, 33, 36]. First, we found that when TEAD1 was knocked down in CAFs, the expression levels of SRC, p-SRC and α-SMA decreased. The phosphorylation of YAP1 was elevated when the YAP1 total protein was unchanged (Fig. 3e). This indicates that once YAP1 was unable to bind to TEAD1, it would exist in a phosphorylated form, thereby losing activity. We knocked down and overexpressed YAP1 and TEAD1 in CAF cells, respectively. The western blot results showed that the knockdown of YAP1 or TEAD1 alone reduced the expression of SRC and p-SRC in CAFs (Fig. 3f). When YAP1 and TEAD1 were simultaneously knocked down, the expression of SRC decreased most significantly. To confirm that YAP1 and TEAD1 act synergistically, we knocked down YAP1 and overexpressed TEAD1 and found that the expression of SRC or p-SRC did not increase significantly. Similarly, when YAP1 was overexpressed after knocking down TEAD1, the expression level of SRC or p-SRC was not compensated (Fig. 3f). In addition, we also observed a positive correlation between the expression levels of α-SMA and SRC. The co-IP experiments confirmed that YAP1 and TEAD1 can form a complex in CAFs (Fig. 3g). To further explore whether the YAP1/TEAD1 complex can regulate SRC transcription, we used JASPAR (http://jaspar2016.genereg.net/) to predict regions where YAP1 and TEAD1 may bind to the SRC promoter region (Supplementary Table 3). According to the score, there are 2 possible YAP1 binding regions and 3 possible TEAD1 binding regions in the promoter region of SRC. The PCR results of the CHIP assay showed that YAP1 did not bind to the promoter region of SRC, while TEAD1 did so. The binding region sequence was “CACATTTCTCCC” (Fig. 3h). Figure 3i shows a schematic diagram of the binding of the YAP1/TEAD1 complex to the SRC promoter region (Fig. 3i). To further examine the regulation of SRC transcription by this protein complex, we performed a dual luciferase reporter assay using 293 T cells. The fluorescence intensity of SRC was not significantly higher than that of the control group which overexpressed YAP1 or TEAD1 alone. Only when YAP1 and TEAD1 were overexpressed at the same time was the fluorescence intensity of SRC significantly increased, indicating that its transcription was activated. Thus, it is believed that YAP1 forms a protein complex with TEAD1 and that TEAD1 binds to the promoter region of SRC. Together, they regulate SRC transcription. Both YAP1 and TEAD1 are indispensable during this process.

Since SRC is known to maintain the CAF phenotype by regulating cytoskeletal proteins and actins, three SRC target genes were selected for further examination [28]. Because SRC as a downstream molecule was regulated by YAP1 / TEAD1, when the SRC in CAF was knocked down, the protein expression levels of YAP1 and TEAD1 were unchanged. However, the downstream gene expression level of SRC was downregulated, and the SMA levels were also decreased (Fig. 3j). Moreover, when YAP1 was knocked down in the CAFs, the mRNA and protein levels of the SRC target gene were also significantly reduced (Fig. 3k-l). Furthermore, when the expression of SRC in the CAFs was reduced by siSRC and its inhibitors, the conditioned medium did not promote the invasion of epithelial cells, and the invasion efficiency decreased significantly (Supplementary Figure S3B-C).

### Fibroblasts with high expression of YAP1 promote tumour growth in vivo

**Fig. 4:**
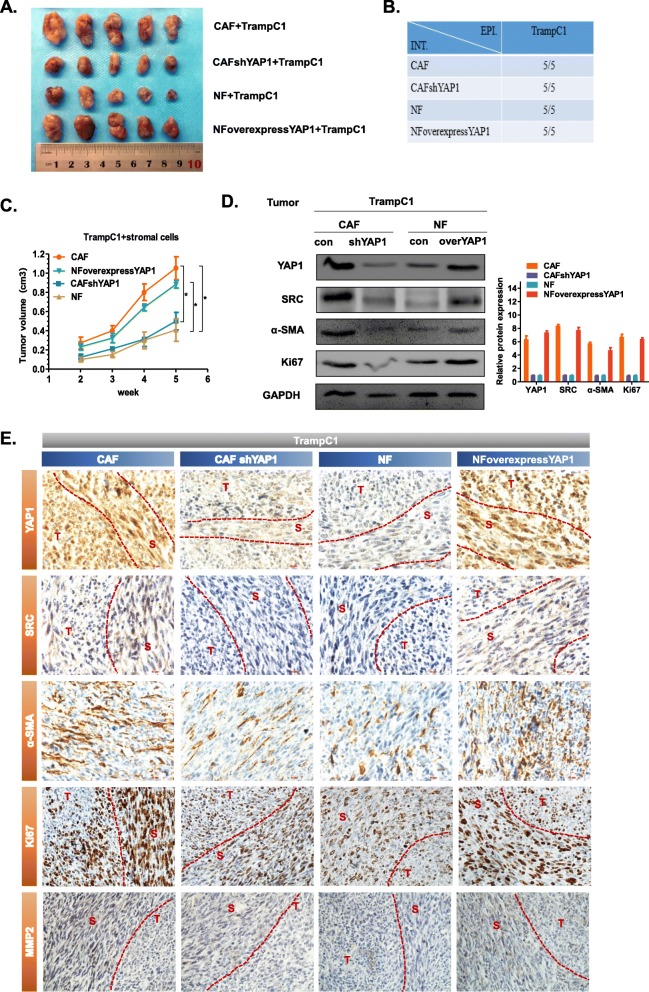
Fibroblasts with high expression of YAP1 promote tumour growth in vivo. **a** Tumour photos taken with a digital camera. **b** Statistical table of the number of tumours in each experimental group. **c** The line graph shows the subcutaneous tumour volumes as a function of time. The data were recorded from the second week to the fifth week (* *P* < 0.05). **e** Western blot was used to detect the expression levels of YAP1, SRC, α-SMA and Ki67 in tumour tissues. GAPDH was used as an endogenous reference gene. **f** Immunohistochemical staining was used to detect the expression levels and positions of YAP1, SRC, α-SMA, Ki67 and MMP2 in tumour tissues. The tumour cells and stromal cells were separated by curves. T: tumour cells, S: stromal cells

In the above studies, we have confirmed that stromal cells, both CAFs and NFs, play an important regulatory role in the proliferation of tumour epithelial cells. Additionally, we showed that when YAP1 expression level was high in stromal cells, NFs will be activated into CAFs, and stromal cells with high expression of YAP1 could significantly promote the proliferation of epithelial cells. For further research, we conducted animal experiments. We mixed each of the four stable cell lines mentioned in the paper before with the epithelial cell line TrampC1 at a ratio of 1:1 to obtain 4 different experimental groups: CAF + TrampC1, CAFshYAP1 + TrampC1, NF + TrampC1, and NFoverexpressYAP1 + TrampC1. Five BALB/c nude mice were in each group. We subcutaneously injected the above-mentioned cells into BALB/c nude mice one at a time [23]. We measured the size of the tumour by Vernier callipers after 2 weeks and counted the number of tumours. The measurement was repeated every 2 days for 3 weeks. Five weeks after the inoculation, the tumours were removed for subsequent experiments (Fig. 4a). We found that the tumour formation rate of the four experimental groups was 100% (Fig. 4b). Comparing the CAF + Trampc1 group to the CAFshYAP1 + Trampc1 group, the tumour growth rate in the CAF + Trampc1 group was significantly faster, and the tumour volume was also significantly larger after 5 weeks (Fig. 4c). Furthermore, the NF + TrampC1 group had a significantly slower tumour growth rate compared with the NFoverexpressYAP1 + Trampc1 group, and the tumour volume was also significantly smaller after 5 weeks (Fig. 4c). This demonstrated that YAP1 indirectly affects the proliferation of epithelial cells through the regulation of stromal cells and that the highly expressed YAP1 stroma cells could effectively promote tumour growth. The results showed that compared to the CAF + Trampc1 group, the expression levels of YAP1, SRC, α-SMA and Ki67 were significantly down-regulated in the CAFshYAP1 + Trampc1 group (Fig. 4d). Moreover, when comparing to the NF + TrampC1 group, the expression levels of YAP1, SRC and Ki67 were significantly up-regulated in the NFoverexpressYAP1 + Trampc1 group. We can distinguish between tumour cells and stromal cells by the morphology of the cells in the IHC picture (Fig. 4e). Curves are used to distinguish tumour cells (T) from stromal cells (S). The specific distinctions were that the tumour cells were small in volume, the cells were mostly round, and the cells are arranged in a disorderly manner; the stromal cells were large in volume, and the cells were fusiform and distributed in a strip-like manner. We found that the protein expression levels of YAP1, SRC, α-SMA, Ki67 and MMP2 in the CAF + Trampc1 group were higher than those in the CAFshYAP1 + Trampc1 group in stromal cells, while the expression levels of YAP1, SRC, α-SMA, Ki67 and MMP2 in the NF + Trampc1 group were lower than those in the NFoverexpressYAP1 + Trampc1 group. More strikingly, the expression of these proteins is also increased when tumour cells are affected by stromal cells. The in vivo experiments confirmed that fibroblasts with high expression of YAP1 could significantly promote tumour growth and were more likely to cause metastasis.

### Expression of YAP1 in the CAFs of prostate cancer patients

**Fig. 5:**
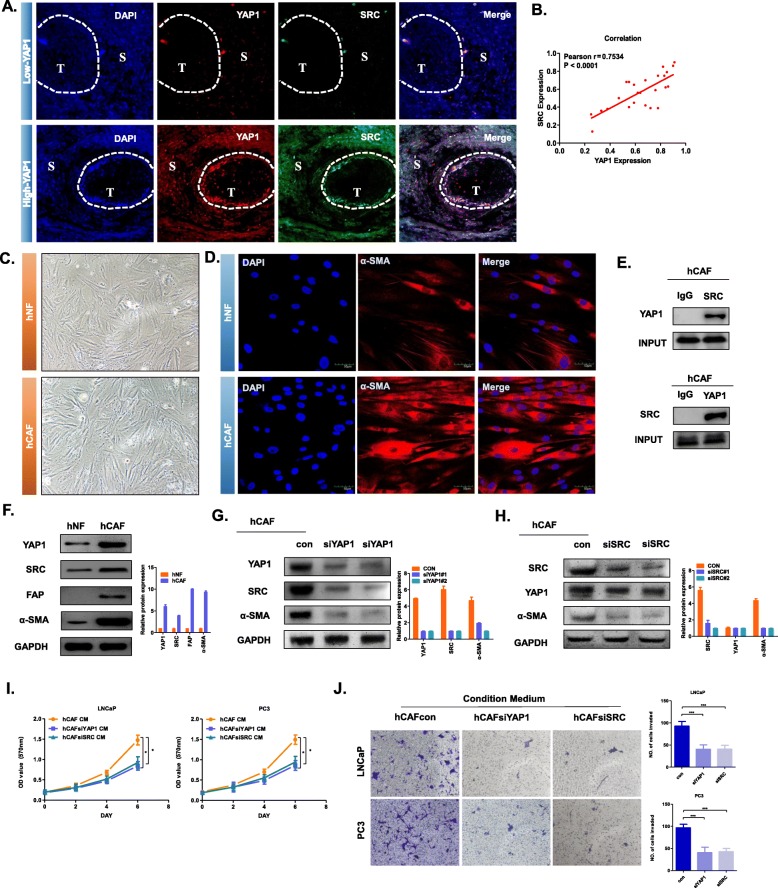
Expression of YAP1 in the CAFs of prostate cancer patients. **a** Immunofluorescence staining for the detection of YAP1 and SRC expression in stromal cells of PCa samples (*n* = 25). The representative images had a magnification of 400 x. **b** Correlation between expression of YAP1 and SRC in prostate cancer stromal cells. The abscissa represents the positive rate of YAP1 in prostate cancer stromal cells, and the ordinate represents the positive rate of SRC in prostate cancer stromal cells. Pearson *r* = 0.7534, *P* < 0.001. **c** Primary cell culture was performed by clinical surgical specimens, and the primary hCAF and hNF cells were photographed under a light microscope. The representative images were magnified 200 x. **d** Immunofluorescence technique was used to detect the expression level and location of α-SMA in hNF and hCAF. The representative images were magnified 400 x. **e** The interaction between YAP1 and SRC in the hCAF was determined by the co-IP assay. The relative levels of YAP1 or SRC in these cells were determined by western blot using a YAP1 or SRC antibody. **f** Western blot was used to detect the protein expression levels of YAP1, SRC, FAP and α-SMA in hNF and hCAF. **g** The protein expression levels of YAP1, SRC and α-SMA were detected after transfection of hCAF with siYAP1. **h** The protein expression levels of YAP1 and α-SMA were detected after transfection of hCAF with siSRC. GAPDH was used as an endogenous reference gene. **i** The MTT experiment showing the effect of the conditioned medium on hCAF cell lines on the proliferation of the epithelial cells LNCaP or PC3. The absorbance value was detected at a wavelength of 570 nm (*P < 0.05). **j** The Transwell invasion assay detects the effect of the conditioned medium on hCAF cell lines on the invasive ability of the epithelial cells LNCaP or PC3. Statistical results (right side) of the above invasive ability. Five visual field counts were taken for each group, and the ordinate indicates the number of invading cells (***P < 0.001)

To more accurately verify the above experimental results, we performed immunofluorescence double staining on the above 25 specimens of prostate cancer. According to the YAP1 expression, we divided the results into two groups, High-YAP1 and Low-YAP1 (Fig. 5a). We distinguished tumour stromal cells from tumour epithelial cells, and we focused on the stromal cell area. Since we have previously demonstrated that once YAP1 was up-regulated on stromal cells, fibroblast would be converted into CAF, so we believed that most of the stromal cells in the “High-YAP1” group were CAF. IF results showed that YAP1 and SRC overlap in large areas of stromal cells (Fig. 5a). This indicated that YAP1 and SRC were co-localized to CAF. By counting the protein expression in the stromal cell region, we calculated the correlation between YAP1 and SRC expression (Fig. 5b). The magnification of the representative picture was 400 times. We can clearly see that most of YAP1 was located in the nucleus and SRC was located in the cytoplasm. Then, we obtained human-derived normal fibroblasts (hNF) and human-derived cancer-associated fibroblast (hCAF) cells from surgical specimens of PCa and BPH patients (Fig. 5c). These cells were identified by their morphology and the protein marker α-SMA (Fig. 5d). Immunofluorescence showed that in hNF and hCAF, α-SMA was distributed in the cytoplasm and that the expression of α-SMA was significantly higher in the hCAF than in the hNF. Co-IP results showed that there was an interaction between YAP1 and SRC in hCAF (Fig. 5e). To verify the changes in the signalling pathways described above, we used western blotting to detect the expression levels of related proteins in hNF and hCAF. The results showed that the expression levels of YAP1 and SRC were significantly higher in the hCAF than in the hNF (Fig. 5f). We knocked down YAP1 in the hCAF using siRNA and found that the expression levels of SRC, FAP and α-SMA were downregulated in the hCAF (Fig. 5g); we also knocked down SRC in the hCAF using siSRC and found that the level of YAP1 did not obviously change in the hCAF but that the expression levels of FAP and α-SMA were significantly downregulated (Fig. 5h). This is consistent with the experimental results described above; namely, in CAFs, the downregulation of YAP1 can reverse CAFs into NFs, in which SRC plays a role downstream of YAP1.

In addition, we examined the effect of hCAF on the proliferation and invasion ability of human prostate cancer cell lines LNCaP and PC3. The results of MTT assays showed that when YAP1 or SRC of hCAF was knocked down, its conditioned medium’s promotion effect on the proliferation capacity of two tumor cells was weakened (Fig. 5i). Not only that, if YAP1 or SRC of hCAF was knocked down, then its conditioned medium cannot promote tumor cell invasion (Fig. 5j). These results showed that hCAF could promote tumor cell proliferation and invasion, and this function depended on the high expression of YAP1 in hCAF.

## Discussion

The TME plays an important role in the occurrence and development of solid tumours. An increasing number of studies have begun to focus on the TME. Cell types in the TME include neuroendocrine cells, fat cells, endothelial cells, mesenchymal cells, immune inflammatory cells, and fibroblasts [37]. The normal fibroblasts are usually quiescent. When their intrinsic signalling pathway is abnormal, they are induced to convert into cancer-associated fibroblasts (CAFs). CAFs are characterized by increased expression of markers such as α-SMA, fibroblast activation protein (FAP), fibroblast-specific protein 1 (FSP1 or S100A4), vimentin and platelet-derived growth factor receptor (PDGFR)-α and β [38, 39]. CAFs can be derived from different cell types, such as NFs, epithelial cells after the EMT, endothelial cells through the endothelial-mesenchymal transition (EndMT), bone marrow-derived cells (BMDCs), adipocytes and stellate cells [40]. The interaction between CAFs and tumour epithelial cells has led to the failure of tumour treatment.

Yes-associated protein was discovered by Sudol et al. in 1994 as a 65 kda protein containing the WW domain [41]. Among many pathogenic proteins, the YAP1 protein functions as a transcriptional coactivator and a connexin [42]. YAP1 is capable of cell proliferation, induction of the epithelial-mesenchymal transition (EMT), enhancement of cell migration/invasion, and inhibition of apoptosis. When YAP acts as a transcriptional coactivator, it needs to interact with the transcription factor TEAD to function. The TEAD protein alone does not induce gene expression and requires additional coactivators to achieve its transcriptional potential [36]. TEAD1 is involved in the regulation of prostate epithelial cell differentiation and epithelial morphogenesis. TEAD1 expression levels are higher in PC3 cells and tissue specimens, which is associated with poor prognosis in prostate cancer patients [43]. SRC is a non-receptor tyrosine protein kinase. Studies have shown that Src protein overexpression and sustained activation are found in solid tumours such as breast cancer [44], colon cancer [45] and pancreatic cancer [46]. In addition, the high activation of SRC is also present in prostate cancer tissues [47, 48]. SRC regulates the expression levels of actin and cytoskeletal proteins in cells [28]. Actin and cytoskeletal proteins are extremely active during the conversion of NFs to CAFs. The link between YAP1 and SRC in prostate cancer specifically indicates that the prostate cancer stroma has not been explicitly implicated. This study demonstrates the role of YAP1, TEAD1 and SRC in the conversion of NFs to CAFs in prostate cancer.

We first found that the expression level of YAP1 in the prostate cancer stroma was significantly higher than that in BPH, and the amount of CAF in the prostate cancer stroma increased with the increase in the YAP1 expression level (Fig. 1). Through the detection of the CAF-specific protein markers SMA and FAP, we found that when the expression of YAP1 in NF was increased, NFs were converted to CAFs. Its function may be similar to that of CAF (i.e., promote the proliferation and invasion of epithelial cells). In contrast, when the level of YAP1 expression in CAFs is lowered, it reverses the above conversion and attenuates the function of promoting proliferation and invasion (Fig. 2). We further confirmed that YAP1 forms a protein complex with TEAD1, which together regulate the transcription of SRC in fibroblasts. SRC regulates the downstream actin and cytoskeletal proteins (such as MYL9, F-actin and paxillin), which ultimately leads to the conversion of NFs to CAFs (Fig. 3). We have demonstrated that fibroblasts with high expression of YAP1 can promote tumour proliferation in vivo (Fig. 4). Clinical features have shown that cancer metastasis occurs almost exclusively in prostate cancer patients who have a high expression of YAP1 in stromal cells (Table 1). Therefore, the high expression of YAP1 may indicate a poor prognosis.

At present, the diagnosis and treatment of prostate cancer is a recognized problem in the world. Abnormal expression of proteins such as PSA and AR has been used as a biomarker for the diagnosis or treatment of prostate cancer. However, some malignant prostate cancers, such as metastatic prostate cancer, do not have an appropriate biomarker as a diagnostic basis. Through our research, we believe that YAP1 in stromal cells has the potential to serve as a diagnostic marker or therapeutic target for prostate cancer. Since we demonstrated that the upregulation of YAP1 in stromal cells leads to the proliferation and metastasis of prostate cancer cells, we draw the following two conclusions: 1. by detecting the expression of YAP1 in the tumour stroma of patients with prostate cancer, we can predict the trend by which prostate cancer develops, and 2. the clinical application of verteporfin (VP) can prevent the proliferation or metastasis of prostatic tumours. In this regard, the research group will continue to conduct in-depth research and strive to use YAP1 as a therapeutic target for prostate cancer. In the follow-up study, the research team continued to explore the effect of tumour epithelial cells on mesenchymal cells. A very real possibility is that the exosomes that are released by epithelial cells are absorbed by mesenchymal cells and that both NFs and CAFs can absorb the exosomes. YAP1 expression levels increase when NFs are affected by exosomal-derived nucleic acids, and then the NFs are converted into CAFs. CAFs also maintain high expression levels of YAP1 due to the influence of related factors from the exosomes, thereby maintaining their own characteristics and functions. In this manner, CAFs will continue to affect epithelial cells, forming a positive feedback loop from the epithelial cells to mesenchymal cells and then back to epithelial cells. This positive feedback loop allows tumours to proliferate. In this feedback loop, YAP1 plays as a decisive factor and is necessary for CAFs to promote tumour proliferation and invasion. We will continue to study this positive feedback loop and determine the mechanism of YAP1 in the tumour microenvironment.

In summary, we confirmed a new mechanism of YAP1 in the prostate cancer stroma. The function of YAP1 was critical for the formation of CAF and maintenance of its own characteristics. Of particular note is that YAP1 is a key factor in converting NFs into CAFs. The YAP1 protein in the tumour stroma can be used as a potential target for tumour diagnosis and treatment. The expression of YAP1 in the tumour stroma may indicate the trend and prognosis of the disease, which provides new ideas and directions for cancer treatment in the future.

## Conclusion

YAP1 can convert NFs into CAFs in the tumour microenvironment of PCa, thus promoting the development and metastasis of prostate cancer.

## Supplementary information

**Additional file 1 Figure S1.** YAP1, α-SMA and FAP are upregulated in CAFs A. The mRNA expression levels of YAP1 and α-SMA in CAFs and NFs were detected by qRT-PCR. B. The protein expression levels of YAP1 and α-SMA in CAFs and NFs were detected by western blot. GAPDH was used as an endogenous reference gene. C. Immunofluorescence staining showed the expression level and location of α-SMA and YAP1 in NFs and CAFs. Nuclei were stained with DAPI. The representative images were magnified 400 x.

**Additional file 2: Figure S2.** YAP1 promotes the proliferation of CAFs A. Western blot was used to detect the protein expression levels of YAP1 and α-SMA after siSRC transfection of CAFs. GAPDH was used as an endogenous reference gene. B. CAFs were treated with vehicle or 10 μM VP for 24 h. The cytoplasmic and nuclear proteins were extracted and measured by western blot. C-D. The MTT assay detected the proliferation of CAFs after YAP1 expression was inhibited by siRNA or VP (10 μM). The absorbance value at a wavelength of 570 nm was detected (*P < 0.05). E. Western blot was used to detect the expression levels of YAP1 and α-SMA after YAP1 was overexpressed. F. The MTT assay detected the proliferation of CAFs after YAP1 was overexpressed. The absorbance value at a wavelength of 570 nm was detected (*P < 0.05)

**Additional file 3: Figure S3.** SRC regulates the invasive ability of epithelial cells through CAFs A. Kaplan-Meier survival analysis of overall survival for SRC expression in PCa. B. Transwell invasion assay for the effect of conditioned medium on the invasive ability of epithelial cells TrampC1 when SRC was inhibited in the CAFs by dasatinib (10 μM) or siRNA. C. Statistical results of the above invasive ability. Five visual field counts were taken for each group, and the ordinate indicates the number of cells invading. (***P < 0.001).

**Additional file 4.**

**Additional file 5.**

**Additional file 6.**

## Data Availability

The data and material that were used or analysed during the current study are available from the corresponding author on reasonable request.

## Abbreviations

AR: Androgen receptor
BPH: Benign prostate hyperplasia
CAF: Cancer associated fibroblast
CHIP: Chromatin immunoprecipitation
Co-IP: Co-immunoprecipitation
FAP: Fibroblast activation protein
GEPIA: Gene expression profiling interactive analysis
hCAF: human-derived cancer-associated fibroblast
hNF: human-derived normal fibroblast
ISUP: International Society of Urological Pathology
NF: Normal fibroblast
PCa: Prostate cancer
SRC: SRC proto-oncogene, non-receptor tyrosine kinase
TEAD1: TEA domain transcription factor 1
YAP1: Yes-associated protein 1
α-SMA: alpha-smooth muscle actin
