# Supplementary figures and images for: YAP1 plays a key role of the conversion of normal fibroblasts into cancer-associated fibroblasts that contribute to prostate cancer progression
Source: J Exp Clin Cancer Res. 2020 Feb 17;39:36. doi: 10.1186/s13046-020-1542-z (PMC7027236; doi:10.1186/s13046-020-1542-z)

Supplementary Figure S1. YAP1,  $\alpha$ -SMA and FAP are up-regulated in CAF

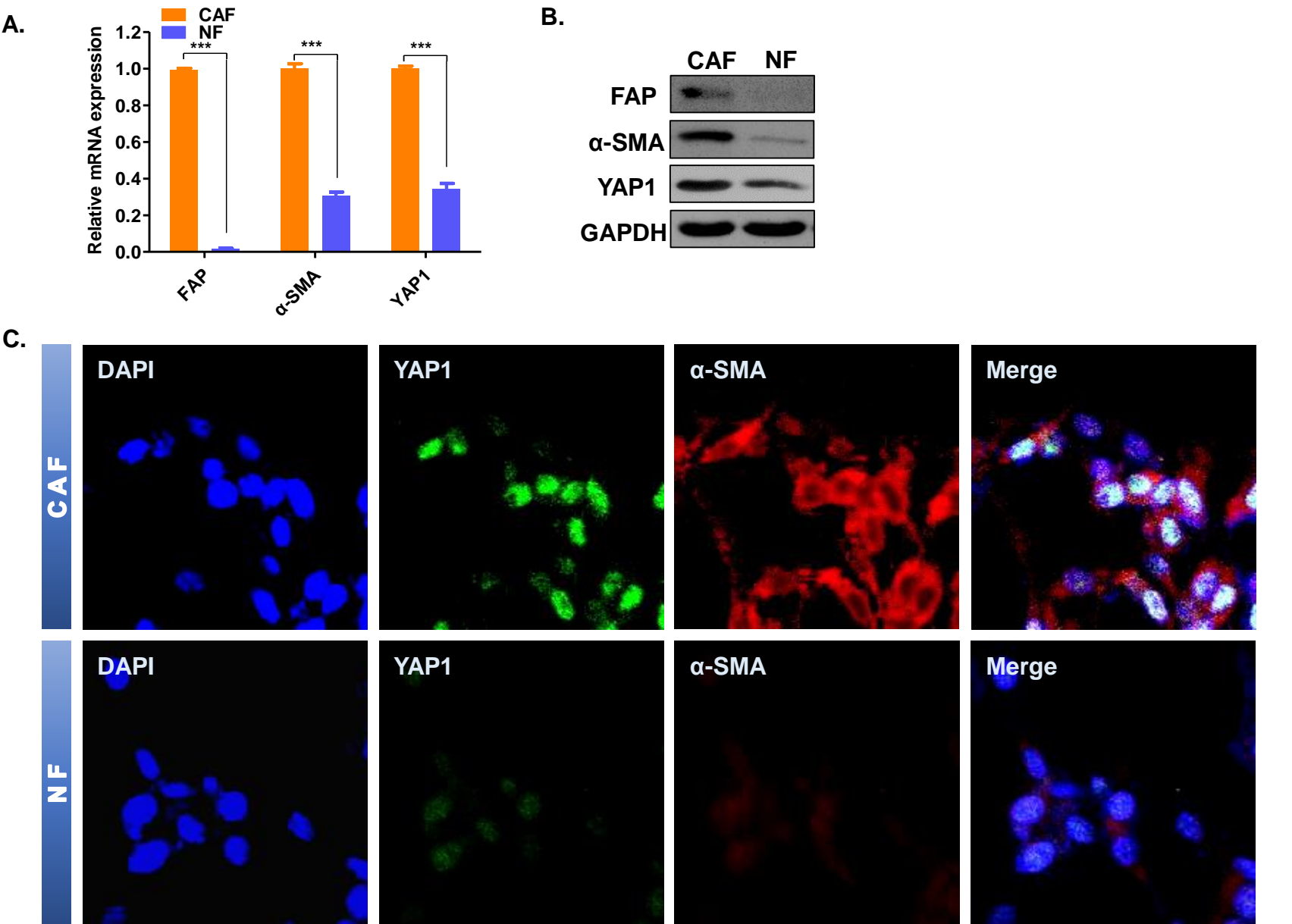

Supplement: Supplementary file 1 — Additional file 1 Figure S1. YAP1, α-SMA and FAP are upregulated in CAFs A. The mRNA expression levels of YAP1 and α-SMA in CAFs and NFs were detected by qRT-PCR. B. The protein expression levels of YAP1 and α-SMA in CAFs and NFs were detected by western blot. GAPDH was used as an endogenous reference gene. C. Immunofluorescence staining showed the expression level and location of α-SMA and YAP1 in NFs and CAFs. Nuclei were stained with DAPI. The representative images were magnified 400 x. [file 13046_2020_1542_MOESM1_ESM.pdf]

Supplementary Figure S2. YAP1 promotes proliferation of CAF

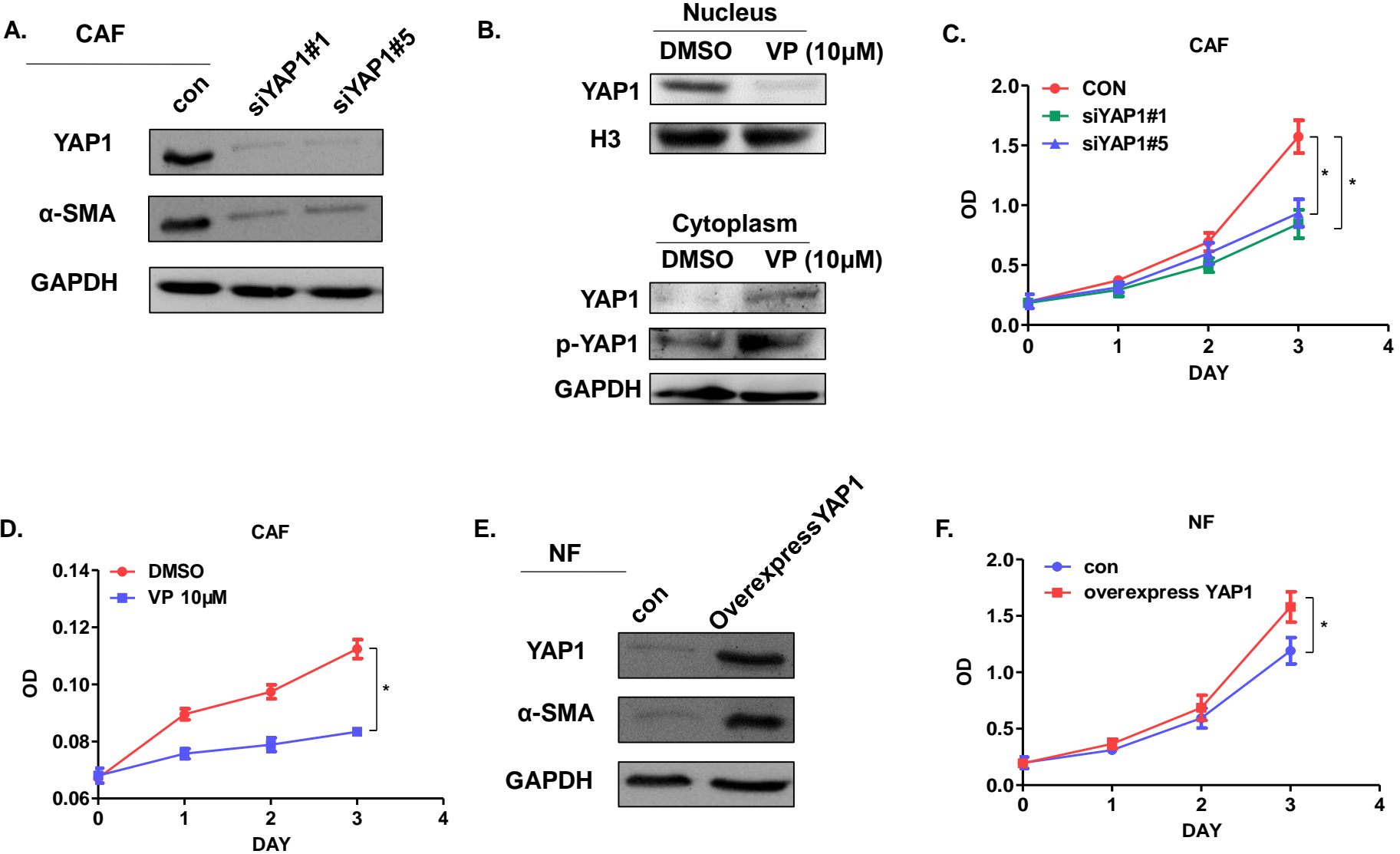

Supplement: Supplementary file 2 — Additional file 2: Figure S2. YAP1 promotes the proliferation of CAFs A. Western blot was used to detect the protein expression levels of YAP1 and α-SMA after siSRC transfection of CAFs. GAPDH was used as an endogenous reference gene. B. CAFs were treated with vehicle or 10 μM VP for 24 h. The cytoplasmic and nuclear proteins were extracted and measured by western blot. C-D. The MTT assay detected the proliferation of CAFs after YAP1 expression was inhibited by siRNA or VP (10 μM). The absorbance value at a wavelength of 570 nm was detected (*P < 0.05). E. Western blot was used to detect the expression levels of YAP1 and α-SMA after YAP1 was overexpressed. F. The MTT assay detected the proliferation of CAFs after YAP1 was overexpressed. The absorbance value at a wavelength of 570 nm was detected (*P < 0.05) [file 13046_2020_1542_MOESM2_ESM.pdf]

Supplementary Figure S3. SRC regulates the invasive ability of epithelial cells through CAF

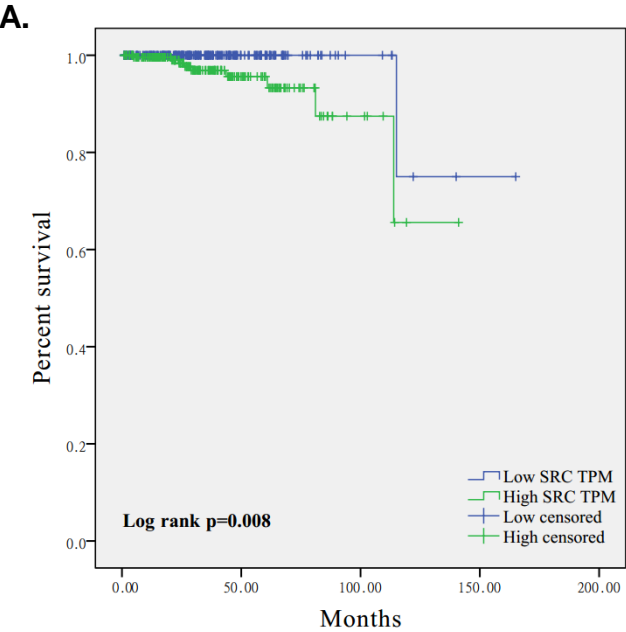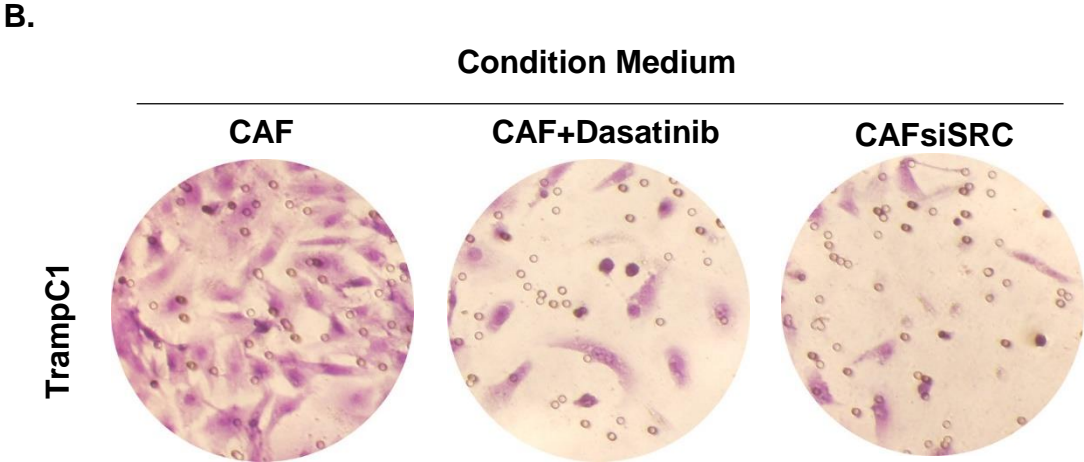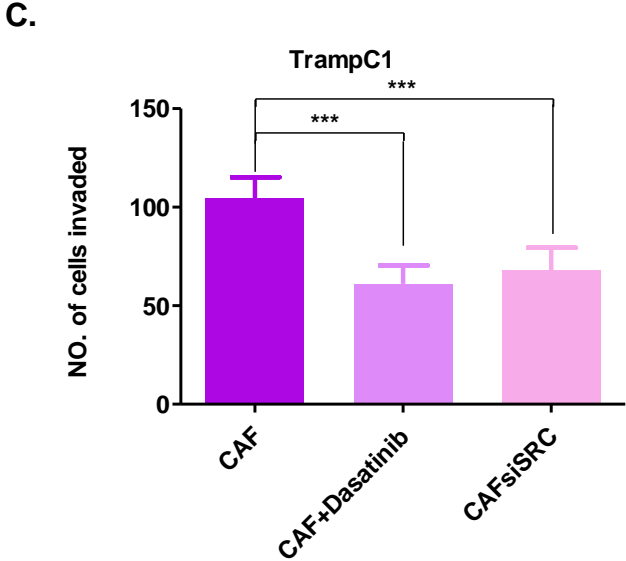

Supplement: Supplementary file 3 — Additional file 3: Figure S3. SRC regulates the invasive ability of epithelial cells through CAFs A. Kaplan-Meier survival analysis of overall survival for SRC expression in PCa. B. Transwell invasion assay for the effect of conditioned medium on the invasive ability of epithelial cells TrampC1 when SRC was inhibited in the CAFs by dasatinib (10 μM) or siRNA. C. Statistical results of the above invasive ability. Five visual field counts were taken for each group, and the ordinate indicates the number of cells invading. (***P < 0.001). [file 13046_2020_1542_MOESM3_ESM.pdf]
