# Supplementary material for: YAP1 plays a key role of the conversion of normal fibroblasts into cancer-associated fibroblasts that contribute to prostate cancer progression
Source: J Exp Clin Cancer Res. 2020 Feb 17;39:36. doi: 10.1186/s13046-020-1542-z (PMC7027236; doi:10.1186/s13046-020-1542-z)
Supplement: Supplementary file 4 — Additional file 4. [file 13046_2020_1542_MOESM4_ESM.docx]

|  | Sequence (5' -> 3') |
| --- | --- |
| siYAP1#1(mouse) | CCACCAAGCUAGAUAAAGA |
| siYAP1#2(mouse) | GGAGAAGUUUACUACAUAA |
| siSRC#1(mouse) | CCGACUUCGACAAUGCCAA |
| siSRC#2(mouse) | CCGUAUGUCCCACAUCCAA |
| siTEAD1#1(mouse) | GCGUCUGGAGUCCUGAUAU |
| siTEAD1#2(mouse) | GGAAUUCUCCGCUUUCCUU |
| siYAP1#1(human) | GGTGATACTATCAACCAAA |
| siYAP1#2(human) | CATTAACGACTAGATTAAA |
| siSRC#1(human) | GGCUCCAGAUUGUCAACAA |
| siSRC#2(human) | GCCUCUCAGUGUCUGACUU |
| siTEAD1#1(human) | GGAUCAGACUGCAAAGGAU |
| siTEAD1#2(human) | CCACUGCCAUUCAUAACAA |

Supplementary Table 1. siRNA Sequence
