# Supplementary material for: YAP1 plays a key role of the conversion of normal fibroblasts into cancer-associated fibroblasts that contribute to prostate cancer progression
Source: J Exp Clin Cancer Res. 2020 Feb 17;39:36. doi: 10.1186/s13046-020-1542-z (PMC7027236; doi:10.1186/s13046-020-1542-z)
Supplement: Supplementary file 5 — Additional file 5. [file 13046_2020_1542_MOESM5_ESM.docx]

|  |  | Sequence (5' -> 3') |
| --- | --- | --- |
| YAP1 | Forward Primer | ACCCTCGTTTTGCCATGAAC |
|  | Reverse Primer | TGTGCTGGGATTGATATTCCGTA |
| α-SMA | Forward Primer | CCCAGACATCAGGGAGTAATGG |
|  | Reverse Primer | TCTATCGGATACTTCAGCGTCA |
| FAP | Forward Primer | GTCACCTGATCGGCAATTTGT |
|  | Reverse Primer | TCGTAGATGTAGTATGTCGCTGT |
| SRC | Forward Primer | TTTGGCAAGATCACTAGACGGG |
|  | Reverse Primer | GAGGCAGTAGGCACCTTTTGT |
| MYL9 | Forward Primer | AGAGGGCTACGTCCAATGTCT |
|  | Reverse Primer | CTCCAGATACTCGTCTGTGGG |
| F-Actin | Forward Primer | TGGATGTCAATCTTGGATGAGC |
|  | Reverse Primer | GCTGGACCGCATACTCTGTG |
| paxillin | Forward Primer | AACGGCCAGTGTTCTTGTCAG |
|  | Reverse Primer | CACCGCAATCTCCTGGTATGT |
| SRC promoter | Forward Primer (TEAD1) | CCCAGACCTTTCCTGTTGA |
|  | Reverse Primer (TEAD1) | GTCCTTCCCTGTGCCATACT |
|  | Forward Primer (TEAD1) | TTACCCAGGATCACGAAGG |
|  | Reverse Primer (TEAD1) | GACGAGGTAGGTTTGCACTG |
|  | Forward Primer (TEAD1) | GGGAGACATAGCTGTACCCTG |
|  | Reverse Primer (TEAD1) | TGTTCCTTCCTTGAAAGTGC |
|  | Forward Primer (YAP1) | TGAGGACTTATCAGTGCAAACC |
|  | Reverse Primer (YAP1) | TCCCCAGAAATTCAACAGGA |
|  | Forward Primer (YAP1) | AATGAAACCCTTGTGCTGG |
|  | Reverse Primer (YAP1) | CGAGGTAGGTTTGCACTGAT |

Supplementary Table 2. Primer Sequence
