# Supplementary material for: YAP1 plays a key role of the conversion of normal fibroblasts into cancer-associated fibroblasts that contribute to prostate cancer progression
Source: J Exp Clin Cancer Res. 2020 Feb 17;39:36. doi: 10.1186/s13046-020-1542-z (PMC7027236; doi:10.1186/s13046-020-1542-z)
Supplement: Supplementary file 6 — Additional file 6. [file 13046_2020_1542_MOESM6_ESM.docx]

Supplementary Table 3 SRC promoter-JASPAR

| 4 putative sites were predicted with these settings (75%) in sequence named**s** | | | | | | | |
| --- | --- | --- | --- | --- | --- | --- | --- |
| Model ID | Model name | Score | Relative score | Start | End | Strand | predicted site sequence |
| MA0415.1 | YAP1 | 5.560 | 0.764128109500321 | 83 | 102 | -1 | GCAGCCTTACATCAGAGGTT |
| MA0415.1 | YAP1 | 4.899 | 0.754750346410373 | 83 | 102 | 1 | AACCTCTGATGTAAGGCTGC |
| MA0415.1 | YAP1 | 7.064 | 0.785465712658085 | 263 | 282 | -1 | ATGACCTAACGTATGCTATT |
| MA0415.1 | YAP1 | 7.194 | 0.787310053356562 | 263 | 282 | 1 | AATAGCATACGTTAGGTCAT |

| 5 putative sites were predicted with these settings (75%) in sequence named**s** | | | | | | | |
| --- | --- | --- | --- | --- | --- | --- | --- |
| Model ID | Model name | Score | Relative score | Start | End | Strand | predicted site sequence |
| MA0090.1 | TEAD1 | 6.768 | 0.762702856192664 | 8 | 19 | -1 | ACCATCCCTCCG |
| MA0090.1 | TEAD1 | 7.413 | 0.779191998171956 | 164 | 175 | -1 | CACAGTCTTACG |
| MA0090.1 | TEAD1 | 10.528 | 0.858825606335514 | 480 | 491 | 1 | CACATTTCTCCC |
| MA0090.1 | TEAD1 | 6.607 | 0.758586961838142 | 499 | 510 | -1 | TACGTTCTACTT |
| MA0090.1 | TEAD1 | 7.239 | 0.774743764521728 | 1131 | 1142 | -1 | CACAGGCCTGGG |

**SRC**

**NM_009271-promoter**

GGAAGCCCGGAGGGATGGTTAACAACAAAGAGTACTCAGAAAGTTCAGTAATGAAACCCTTGTGCTGGGTGGTTCTTCTGAGAACCTCTGATGTAAGGCTGCCCTCAAGGAGGGCACGTGTGAGGGAGGCTGTGTCTGCAGCAGAGCCAACAGCACTCTTCCACGTAAGACTGTGAGGACTTATCAGTGCAAACCTACCTCGTCTCTCTCATGGAAGCCTTACCCACTGGGCAGGCTGCTGGCATACTGCGGCTCAGGGGGAAATAGCATACGTTAGGTCATGCATATATTTGCACAACGGTGGAGCTCCGGGTTCAACCTTAGCCTGTCAGTCTAAAGTTTATGCTTTGAAGCTCTCTGTCTTCTCAGCTCTAGAACCACCCAGACCTTTCCTGTTGAATTTCTGGGGACAGATGTAGTGATACATTTGGCAAATAGTTAACCAACTCTCTGGAAGGAAAAACTAATGGGTTCATTGTCACATTTCTCCCCATTTCCAAGTAGAACGTACTTCCTCTGAGGCTGACACCAGGTGACCAGTCTGACATCACCAAGTATGGCACAGGGAAGGACACTGCTCAGTAAGTGCTTGCAAGCCGTTTGAGAGCCAGCCCCAGTGCCTCACAGGAAGAGTATCCACCCTCCCCTCCCCTCCCTCAGCCCAGAGTGGCCAGTAAAGGCCATTAGACAAATCTTAGGCATGGAGGAGGGCGGAGAAACCTGCTCCTCCAGCCAGAGCCTGCCCAGGGGAGCCCTGGGAAACTGCCGTTAATCTTTAAGCAAGCCCTGCGAACAGGAGTGGCTGGCTAGTTTGCCCGCTGTTCATGAGAAACCCTGAGCAGTCCCCGTGGCCACAGTGGGCTACCCTGGCCCCACTTGCTCTTGTCAGTGAGGCCCTCAGATCTTCAGCCTGGCCCACTCCTTCTTCACAACAGAGGGGGCTGGAACAGGAAGCTAGTGGACAGAACACACCCTGAGGCACCTGGCCTAGATGTGACCTCAGATCAGTCTGACTCAAAGCTTTGCCCTTTCCTGGTCTCCTGGGAGACATAGCTGTACCCTGGGGCTGGAGCCTCTCACAATCTAGGATGATGGGGGACTTGCCCTTTGGGAGGGATCTTTGCCAGAAGCCCAGGCCTGTGCCACATGAACCTCAGTTTCCCTGTCTGTGAGATGGAAGCACTTTCAAGGAAGGAACATGGAGATGGTTGATAGATGGTCAGGCCCATCTTTCCAGAGTGAACGGGAGGAGTCAAGGATGCTCCTGGACCCCCAACGGGCAGCGCTAGAACCTGGTGGTCTTTGAGACCAGCCTGAAGCCACATCTCATCCCGCTTGCCCCATTTTTCTTCTCTACTTCCTGTCCGGTTGGTTCTGTCATCCTCAGTGGCTGGCAACAAGCACACTGCCCAGCAGATGCACACGGTCTCCTTATGCATTGAAAGTGAATTTAGGCTTGGCCTAATTGAGCTGGGCCCCGCAAGTTCCTGCCCTCCATTGGCAGAGGCTGGAGGCTCCGCAAGTTCAAGGCCAGCCTTGGCCACATAGTGAGCCACTGTTTTGCCTTTTGATACATTTTAACAATACTGCCTCTCCCCCTCACTATTTCCCTGTCCCTTCGGTTCCAGTCTCTGAGGATTAGAGACACTCAGGGTCAAGGCTGGTGTGGCCAGTGCCTCAGTTTCCGGACAGTGCTAGCCACCGGTGCGTAGCTCAGGTAGCTAGGGTTGAACGCTAACGTGGTCCCGGGAGGCCGAGCCCGGCGCGTGGCGTGGGGTTGGGGGGGCGCCCCCGCGGGCGGAGCTGCGGGGGGTGGGGGGCTGGGTGCGGGAGGCGCGGGCCCGGCTCGCACAGCAGGTCTCCCGGGTCGCCGGCGGGGCCCGGGTCCGCGCTTCCTCCTTTCTCCGGCTCGCGGCGGCCGCGGCCCCTCCTCCCGCGCGCTCCCTCCCCCTCGCCGCCGCCGCCGCCACCGCCGCCGCCTCCTCCCTTTCTCTCGGTCTGTCTCTCCGGGCCCGGAATCCAACCGGCCG
